# Prevalence of Varicose Veins Among Nurses at Different Departments in a Single Tertiary Care Center in Riyadh

**DOI:** 10.7759/cureus.12319

**Published:** 2020-12-27

**Authors:** Bader AlBader, Asma Sallam, Afaf Moukaddem, Kout Alanazi, Sara Almohammed, Haya Aldabas, Shahad Algmaizi

**Affiliations:** 1 Family Medicine, King Abdulaziz Medical City, Riyadh, SAU; 2 Medicine, College of Medicine, King Saud bin Abdulaziz University for Health Sciences, Riyadh, SAU; 3 Medical Education, King Saud Bin Abdulaziz University for Health Science, Riyadh, SAU

**Keywords:** varicose, heavy, long-standing, nurses, lifestyle

## Abstract

Background

Varicose veins (VV) is a chronic venous disease that affects the lower extremities.^ ^It is the dilation of subcutaneous veins, three to four millimeters in diameter. VV can be attributed to several risk factors such as age, obesity, multiple parities, heavy lifting, and long-standing hours. Direct and indirect complications can occur due to VV. Several studies were conducted to estimate the prevalence of VV.

Methods

A self-administered questionnaire was used to estimate the prevalence of VV among nurses from different departments in the National Guard Health Affairs (NGHA), Riyadh, Saudi Arabia. The questionnaire included questions on weight, height, work history, lifestyle, and multiple diseases. The chi-square test and Fisher’s exact test were used for testing the association between the various predictors and the diagnosis of VV.

Results

A total of 366 nurses participated in the study. There were 40 (39 females and one male) cases of VV accounting for 11.0%. Variables that have a statistically significant association with VV were social status and lifting heavy objects (p=0.02), a family history of VV (p-value=<0.001), and the number of childbirths (p=0.04). The observation of each department was not statistically significant with VV (p=0.35).

Conclusion

Among nurses, positive family history, age, marital status, long-standing hours, and heavy object lifting were significant risk factors for the development of VV. However, the prevalence of VV in the four departments was low.

## Introduction

Varicose veins (VV) is a chronic venous disease that affects the lower extremities. It is a dilation of 3 to 4 mm in diameter of the subcutaneous veins [1-2]. Several patterns of this disease exist such as reticular, telangiectasia, and trunk veins [3]. The disease can cause various symptoms such as throbbing, swelling, aching, night cramps, and leg fatigue [3-4]. The VV burden can impact the quality of life of affected patients psychologically, physically, and financially [5-7]. It can lead to low self-esteem and social isolation, consequently increasing the risk of depression [5-6]. Easy leg fatigability can interfere with patients’ daily activities and decrease their work productivity [5]. Furthermore, procedures for this disease and its complications may increase the financial burden on the patients [7]. The exact cause of the disease is not clearly understood. However, VV can be attributed to a number of risk factors such as obesity, age, multiple parities, pregnancy, heavy lifting, and long-standing hours [8-10]. VV can result in direct complications, such as thrombophlebitis and hemorrhage, and indirect complications, including skin pigmentation, lipodermatosclerosis, atrophie blanche, edema, varicose eczema, and venous ulceration [3]. VV is diagnosed by a complete history and detailed physical examination followed by accurate diagnostic investigations to determine the nature and extent of venous insufficiencies such as duplex Doppler test, duplex ultrasound imaging, thermography, phlebodynamometry, angioscopy, or capillaroscopy [11]. Ultrasound tests using the Doppler technique are more commonly performed and are considered effective in assessing blood flow dynamics and pathological reﬂexes and obstructions as well as in providing early information about the anatomical state with accurate mapping of abnormal venous pathways [11]. Several modalities of treatment are currently available. Some are invasive such as surgical ligation while others are less invasive such as endovenous laser therapy, ultrasound-guided foam sclerotherapy, and radiofrequency ablation [12-13].

Internationally, studies estimating the prevalence of VV are numerous. One population-based study conducted in Russia on the prevalence of chronic venous disease (CVD) reported that 29% of individuals (31.5% of men, 27.5% of women) were found to have primary varicose veins [14]. In a study done in Brazil, 47.6% (50.9% non-pregnant women and 37.9% men) were found to have the disease [15]. Another VV study involving physicians in Taiwan suggested that long-standing hours do not affect the development of VV in physicians and non-physicians [16]. Also, a study done in western Jerusalem estimated the population prevalence of VV to be 29% among females and 10% among males. Prevalence increased with age and gender to reach 54% among females between 65-74 years of age and 39% among males older than the age of 75 [17]. In Saudi Arabia, a study was done in Jeddah, Dammam, Makkah revealed that the prevalence of VV among females from the general population was 66% [18]. Another study done in Abha investigated the prevalence of VV among school teachers of both genders and reported a high prevalence of 42%; most of them were female teachers [19].

Furthermore, studies involving nurses as a high occupational risk group for VV were found among many different nations. An Iranian study estimated a 72% prevalence among female nurses [20]. Another Korean study found that prolonged standing during work was strongly associated with VV incidence regardless of younger age and short practice duration with an estimated prevalence of 16.18% [21]. A study done in India found that 24.17% of participated nurses had lower limb VV with a slight difference between both genders (24.50% in females v/s 22.58% in males) [22].

In Saudi Arabia, few studies described the prevalence of VV among both sexes of the population, but there were not many studies that described the association between VV and the type of occupation or the number of hours sitting or standing at work and lifestyle factors such as smoking and exercising [18-19,23].

In this study, we aimed to estimate the prevalence of VV among nurses in a large Saudi tertiary care center as well as to determine the occupational and demographic predictors of having VV diagnosis among those nurses. Knowledge of the prevalence of VV in different departments is valuable in assessing the association of the prolonged sitting and standing times and the shifts’ duration with the prevalence of this disease.

## Materials and methods

Study design, area, and settings

This study was a cross-sectional study using a self-administered questionnaire among nurses working at King Abdulaziz Medical City (KAMC), a large tertiary care center in Riyadh, Saudi Arabia. The data were collected from nurses working in four departments: dermatology, intensive care unit, general surgery, and emergency. These departments were chosen due to the differences in the workload between them and the variation in the number of hours spent in standing and sitting positions as well as the number of shifts per week and the magnitude of heavy object handling.

Identification of study participants

The study included nurses from both genders working at KAMC from different nationalities. The study also included all employed nurses whether in administrative jobs or clinical work. Pregnant nurses were excluded from this study. The sample size was calculated using the Raosoft online calculator with a population size of 3340 nurses, a margin of error of 5%, a prevalence of 50%, and a confidence interval of 95%. The sample size was calculated to be 345 nurses.

Data collection process

A self-administered questionnaire was developed by the research team. The questionnaire was validated by a panel of experts specialized in vascular surgery and plastic surgery. It was pilot tested among 28 nurses working in the internal medicine department at KAMC. The clarity of the questionnaire was modified according to the pilot. The questionnaire included sections on demographics (age, gender, ethnicity), behavioral characteristics (smoking, daily exercise), constitutional characteristics (body mass index (BMI), height), work-related variables (sitting and standing periods during work hours and handling heavy objects), which were assessed subjectively by the participants. The questionnaire also included health-related variables (comorbidities, receiving hormonal therapy/contraceptives, the number of gravidas/parities, etc.).

The main outcome that the study measured was the prevalence of diagnosed VV, which was self-reported. Our focus was on assessing the effect of occupational factors and related comorbidities on having this disease among our population.

Data analysis

Data were analyzed using the Statistical Package for the Social Sciences (SPSS), version 21.0 (IBM Corp., Armonk, NY). Descriptive statistics were used to describe the data. Categorical variables, such as gender and ethnicity, were described as frequencies/percentages. Quantitative variables, such as weight, height, and the number of childbirths were described as mean±SD (standard deviation). The chi-square test or Fisher’s exact test were used as applicable to assess the association between the outcome and any categorical variable. To test the association between the outcome and any continuous variable, the student’s t-test was used. The p-value for statistical significance was declared as 0.05.

## Results

A total of 366 nurses participated in the study, including 322 female nurses (88.0%) and 44 male nurses (12.0%). The mean age was 36 years (SD=8), and the male to female ratio was 1:7. Of these, there were 40 (11%) cases with a known diagnosis of VV (39 females and one male). The number of nurses working in the emergency department was 66 (18.0%), in the ICU was 207 (56.4%), in the surgical department was 88 (24.0%), and in the dermatology department was six (1.6%). The average number of years of working as a nurse was 10 years (SD=6), and the average number of shifts per week was 4.8 days (SD=2.8). The average number of hours working in a sitting position was three hours per day (SD=2) while the average number of hours working in a standing position was 8.9 (SD=2.2). The mean BMI of nurses who participated in this study was 24.88 (SD=4.69) (Table 1).

**Table 1:** Characteristics of the study sample (N=366) ICU: intensive care unit; VV: varicose veins; BMI: body mass index

Variable	Frequency	%
Gender		
Female	322	88.8
Male	44	12
Ethnicity		
Arabian	34	9.4
Asian	311	86.1
African	10	2.8
Others	6	1.7
Social		
Single	158	43.8
Married	191	52.9
Widow/Widower	4	1.1
Divorced	8	2.2
Department		
Dermatology	6	1.6
ICU	207	56.4
Surgery	88	24
Emergency	66	18
Smoking		
No	337	91.8
Yes	30	8.2
Exercise		
None	95	26.2
<150 min	171	47.2
>150 min	96	26.5
Diagnosis of VV		
No	324	89
Yes	40	11
Age (Mean, SD)	36.8	
Years working in the field	10.6	
(Mean SD)		
Shift per week (Mean, SD)	4.8, 2.8	
Standing hours (Mean, SD)	8.9, 2.2	
Sitting hours (Mean, SD)	3,2	
BMI (kg/m2) (Mean, SD)	24.88, 4.69	

Concerning VV risk factors, bivariate associations revealed a statistically significant association between the disease and social status (p=0.02), lifting heavy objects (p=0.02), positive family history of VV (p-value=<0.001), number of childbirths (p=0.04), and menopause (p=0.003). Having comorbidities such as hypertension and diabetes has not shown a statistically significant association with the disease but, rather, they appeared somehow as protective factors with p=0.04 and p=0.01, respectively (Table 2). Ethnicity, gender, smoking, regular exercise, using hormonal therapy, and type of delivery were not significant predictors of VV (p>0.05). The variation in the working nature of each department did not show a significant association with VV diagnosis (p=0.35). Among the four departments, the highest prevalence of VV was found among nurses working in the ICU (52.5%), followed by those working in the surgery department (22.5%) and emergency department (20.0%), with the lowest prevalence found in the dermatology department (5.0%) (Table 2).

**Table 2:** Association of various categorical predictors with the diagnosis of varicose veins among nurses (N=366) ICU: intensive care unit; VV: varicose veins

Variable	With VV	Without VV	p-value
	Frequency (%)	Frequency (%)	
Gender			
Women	39 (97.5)	280 (86.7)	0.07
Men	1 (2.5)	43 (13.3)	
Ethnicity			
Arabian	3 (7.5)	31 (9.7)	
Asian	34 (85)	274 (86.2)	0.51
African	2 (5.0)	8 (2.5)	
Others	1 (2.5)	5 (1.6)	
Social status			
Single	14 (35.0)	143 (44.8%)	0.02*
Married	22 (55.0)	168 (52.7%)	
Widow/Widowed	0 (0.0)	4 (1.3%)	
Divorced	4 (10.0%)	4 (1.2%)	
Department			
Dermatology	2 (5.0%	4 (1.2%)	
ICU	21 (52.5%)	185 (57.1%)	
Surgery	9 (22.5%)	77 (23.8%)	0.35
Emergency	8 (20.0%)	58 (17.9%)	
Lifting heavy objects			
No	6 (15.4%)	96 (29.9%)	0.02*
Yes	32 (82.1%)	225 (70.1%)	
Smoking			
No	36 (90.0%)	299 (92.3%)	0.54
Yes	4 (10.0%)	25 (7.7%)	
Exercise			
None	12 (30.8%)	83 (25.9%)	
<150 min	16 (41.0%)	154 (48.1%)	0.69
>150 min	11 (28.2%)	83 (25.9%)	
Family History			
No	15 (38.5%)	229 (71.3%)	<0.001*
Yes	24 (61.5%)	92 (28.7%)	
Deep Vein Thrombosis			
No	37 (94.4%)	316 (98.4%)	0.17
Yes	2 (5.1%)	5 (1.6%)	
Coronary Artery Disease			
No	35 (92.1%)	267 (84%)	
Yes	3 (7.9%)	51 (16%)	0.17
Hypertension			
No	27 (67.5%)	159 (49.8%)	0.04*
Yes	13 (32.0%)	160 (50.2%)	
Diabetes			
No	32 (86.5%)	205 (64.1%)	0.01*
Yes	5 (13.5%)	114 (35.6%)	
Occupational injury			
No	34 (91.9%)	302 (94.4%)	0.47
Yes	3 (8.1%)	18 (5.6%)	
Hormonal therapy			
No	37 (94.9%)	309 (96.6%)	0.64
Yes	2 (5.21%)	11 (3.4%)	
Contraceptive pills			
No	38 (95.0%)	259 (80.7%)	0.07
Yes	1 (2.5%)	19 (5.9%)	
Menopause			
No	31 (81.6%)	269 (95.7%)	0.003*
Yes	7 (18.4%)	12 (4.3%)	
Number of childbirths			
No	37 (94.4%)	279 (99.6%)	0.04*
Yes	2 (5.1%)	1 (0.4%)	
Type of delivery			
Vaginal	14 (70.0%)	61 (47.3%)	0.28
C-section	4 (20.0%)	51 (39.5%)	
Both	2 (10.0%)	16 (12.4%)	

In contrast to nurses without a known diagnosis of VV, those who had been diagnosed with VV were older (mean 41 vs. 36 years, [SD] = 8 years) (p-value <0.001) and have been working in the field for a longer period (mean 14 vs.10 years, [SD] = 6 years) (p-value <0.001). The number of hours of working in a standing or sitting position, number of shifts per week, number of childbirths, and BMI were not significant predictors of VV (all with p>0.05) (Table 3).

**Table 3:** Association of various continuous predictors with the diagnosis of varicose veins among nurses VV: varicose veins; BMI: body mass index

Variable	With VV	Without VV	p-value
	Mean; SD	Mean; SD	
Age	41; 8	36; 8	<0.001*
Number of children	2; 1	2; 1	0.3
Years working in the field	14; 6	10; 6	<0.001*
Shifts per week	5.3; 3.7	4.7; 2.7	0.23
Standing hours	9.4; 1.9	8.8; 2.2	0.09
Sitting hours	3; 2	3; 2	0.21
Number of gravidas	2; 1	2; 1	0.79
Number of parities	2; 1	2; 1	0.84
Number of abortions	0; 1	0; 1	0.96
BMI	25.10; 4.26	24.89; 4.76	0.81

## Discussion

According to our findings, there was no significant difference in gender predisposing to VV. Female nurses were found to have a higher VV prevalence than male nurses, accounting for 97.5% of the total affected population. However, these results are not valid and cannot be concluded since there was a considerable difference in the number of participating nurses from both genders. Sharif Nia’s study considered VV as having a gender-related risk where females are more prone to suffer from the condition [20,24-25]. The female gender preponderance can be explained by being pregnant, in which there is an increase in progesterone levels and uterine wall expansion, which increases blood volume leading to valve failure [24-25]. Our results also showed an association between VV and social status, which might be explained by the fact that marriage can be an indirect risk factor for VV in females due to multiparity [20]. An increase in years of service and lifting heavy objects are considered risk factors for VV owing to the fact that the increase in pressure on the lower limbs leads to vascular wall and valvular damage [20,24-25]. That finding of Tabatabaeifar was concordant with our study, which related the prolonged lifting of heavy objects to VV and an increased risk to surgical treatment [26]. Moreover, Abdel Moneim and Chen were concordant with our findings, where it was observed that higher working years were significantly associated with the presence of VV [23,25]. Also, we have a high percentage of our participants from the ICU department, affected with VV, who are exposed to increased years of service and long-standing hours at the patient's bedside. In addition, our data showed a significant association between VV and family history, which supports Laurika JO et al.’s study but there was no clear genetic pattern found [27]. In our study, there was no significant relationship between hypertension and VV in contrast to other studies in the literature, which relate the disease to the high blood volumes and wide vessels [28]. Also, our study showed no association between diabetes and VV, which is inconsistent with other findings showing a correlation between the two conditions [29]. These discrepancies between our results and other studies showing the association of these comorbidities (hypertension, diabetes) to VV might be explained by the fact that our population are nurses, who are educated and most probably follow a healthy diet and exercise regularly. Moreover, the majority of our participants were nonsmokers and were middle-aged with a normal BMI of less than 25, consequently affecting the overall VV pathogenesis and suggesting a more trend towards the occupational factors as stronger predictors for the formation of VV in this specific population. Also, taking into account the average age of the participated nurses, these comorbidities might be sub-clinical or not yet diagnosed.

Concerning women’s health, our findings reveal a high frequency of VV in menstruating females, which can be related to the high progesterone and estrogen levels. This is discordant with another study that found menopause as a risk factor for VV. This difference may be due to the prominence of middle-aged female nurses who are not yet exposed to menopause with a mean age of 41 years old [30]. According to H Sharif et al., pregnancy is another risk factor for VV owing to increased progesterone levels and vasodilation of blood vessels with valvular failure as well as to the uterine wall expansion, which increases the pressure on the pelvic and lower limb vessels [20,23]. However, our findings showed a significant association between not being pregnant and suffering from VV due to a decreased number of parity and the presence of other predisposing factors. K Kroger’s study suggested that older age is not a direct risk factor of VV, but age-related increased BMI can be [28]. Our analysis showed a statistically significant association of the middle-aged group to VV, which conflicts with K Kroger’s study attributing this difference to the presence of other factors such as family history, long-standing, etc. [28].

Limitations

This study might not be representative of the prevalence of VV in Riyadh since this is a single-center study. The use of a survey-based study without any measurement taken to confirm the diagnosis limited our ability to assess the varices. Moreover, the diagnosis of VV was based on self-report, which might be a source of bias.

## Conclusions

The outcomes of this study reveal a low prevalence of VV among nurses working within four different departments; the variation in the practicing nature of each department has shown no significant association. According to the findings, there is a correlation between the disease and age, social status, positive family history of the disease, lifting heavy objects, and having a longer nursing practice period.

High prevalence in specific departments might necessitate raising awareness and prevention strategies. For subsequent studies, we recommend combining the survey with physical examination and appropriate imaging modalities to confirm the diagnosis and assess the intensity, increasing the number of departments, and including and comparing more than one hospital.
